# Editorial: Regulation of Fruit Ripening and Senescence

**DOI:** 10.3389/fpls.2021.711458

**Published:** 2021-07-26

**Authors:** Carlos R. Figueroa, Cai-Zhong Jiang, Carolina A. Torres, Ana M. Fortes, Noam Alkan

**Affiliations:** ^1^Institute of Biological Sciences, Campus Talca, Universidad de Talca, Talca, Chile; ^2^Crops Pathology and Genetics Research Unit, USDA-ARS, Davis, CA, United States; ^3^Department of Plant Sciences, University of California, Davis, Davis, CA, United States; ^4^Horticulture Department, Tree Fruit Research and Extension Center, Washington State University, Wenatchee, WA, United States; ^5^Faculty of Sciences, BioISI - Biosystems and Integrative Sciences Institute, University of Lisbon, Lisbon, Portugal; ^6^Department of Postharvest Science, Agricultural Research Organization (ARO), Rishon LeZion, Israel

**Keywords:** fruit ripening and senescence, hormonal regulation, cell wall-modifying enzymes, transcription factors, molecule signaling, exogenous molecule application, microbial interaction, postharvest fruit quality

Fruit ripening and senescence comprise complex and highly coordinated molecular and biochemical processes involving ripening-associated genes, transcription factors, enzymes, repressors, signaling molecules, and metabolic pathways in both climacteric and non-climacteric fruits (Cherian et al., 2014; Fuentes et al., 2019), which account for fruit quality on one hand and post-harvest losses on the other. Therefore, studying the molecular mechanisms of fruit ripening and senescence have profound commercial implications. As the fruit ripens or enters senescence, it becomes susceptible to fungal pathogens (Alkan and Fortes, 2015), while fruit-pathogen interactions could accelerate ripening and senescence, resulting in fruit deterioration. Hence, common strategies to slow down senescence and preserve fruit quality include both pre- and post-harvest management practices and technological tools.

This Research Topic aimed to study and characterize the endogenous molecular and biochemical regulators (i.e., hormones, molecules, and genetic components) and their mechanisms of action to regulate ripening, senescence, and disease resistance in fruit. This collection includes 10 original research articles reporting new information on hormonal control of fruit ripening (Upadhyay et al.; Khaksar and Sirikantaramas; Fresno and Munné-Bosch), the effect of exogenous application of signal molecules in post-harvest fruit quality (Yu et al.; Yao et al.; García-Pastor et al.), genetics studies of the fruit cell wall and texture modification-related enzymes (Wen et al.; Nakano et al.), antioxidant-related proteomic changes during ripening (Song et al.), and the effect of mutations of key transcription factors on fruit quality traits (Adaskaveg et al.) (Figure 1). These studies included various fruit species such as tomato, peach, sweet cherry, strawberry, pomegranate, and durian. Moreover, two important reviews on the role of alternative oxidase (Hewitt and Dhingra) and sugar signaling (Durán-Soria et al.) during ripening were included in this Research Topic (Figure 1).

**Figure 1:**
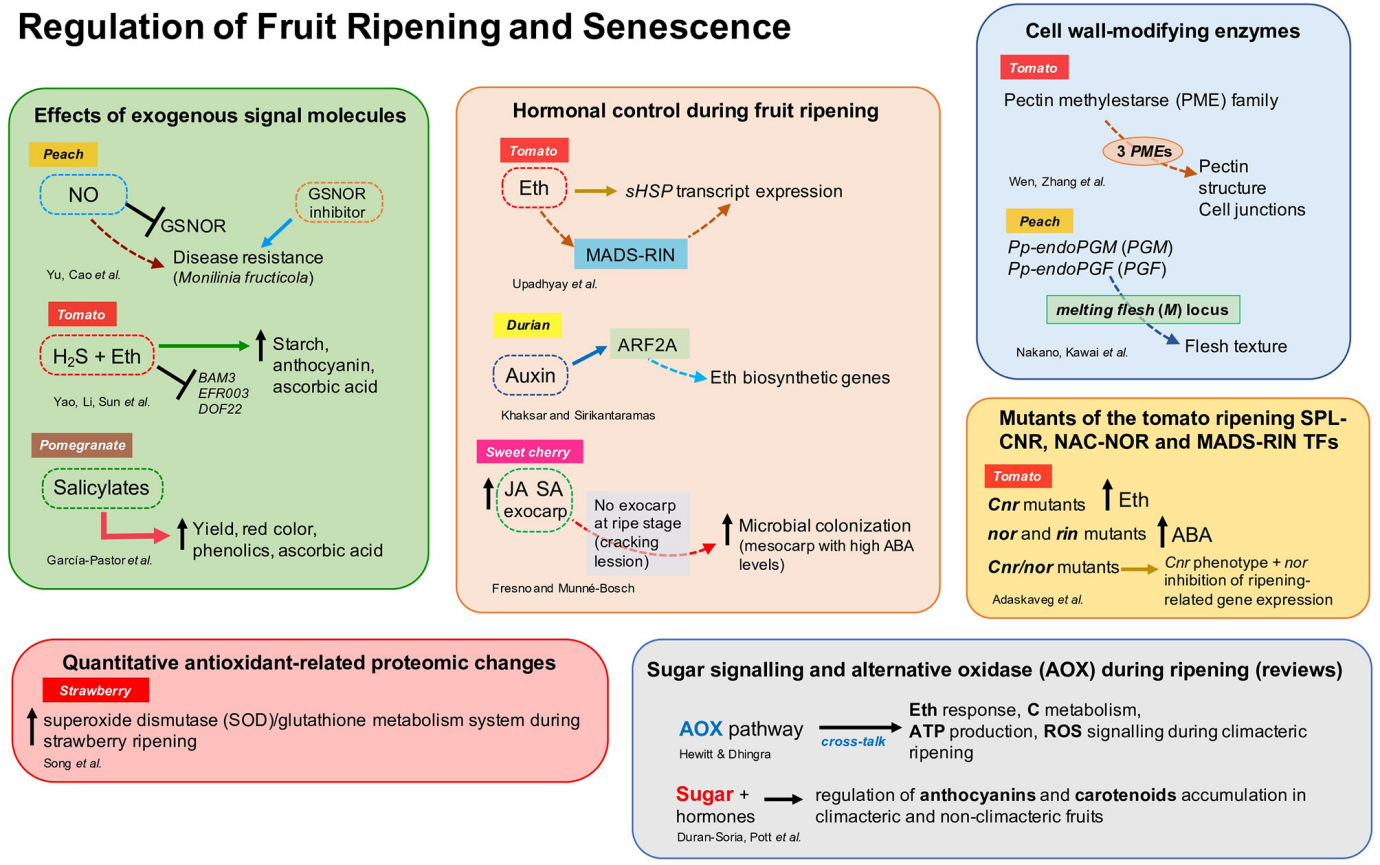
Overview of the different aspects addressed by authors in the Research Topic “Regulation of Fruit Ripening and Senescence.” ABA, abscisic acid; ARF, auxin response factor; Eth, ethylene; GSNOR, S-nitrosoglutathione reductase; H_2_S, hydrogen sulfide; JA, jasmonic acid; NO, nitric oxide; SA, salicylic acid; sHSP, small heat-shock proteins; for other abbreviations, see the respective references.

Upadhyay et al. investigated the regulation of two small heat shock proteins (sHSP) by ethylene and the inhibitor of ethylene receptor 1-methylcyclopropene (1-MCP) during tomato fruit ripening (SlHSP17.7A and SlHSP17.7B). This study showed that a transgenic tomato line silenced in one of the ACC synthase genes (*SlACS2*), whose fruit produced 50% less ethylene, had higher expression of both *sHSP* genes at the transition stages [breaker (BR) and BR+3 days] compared to control fruit. Moreover, the expression of *SlHSP 17.7A* and *SlHSP 17.7B* were significantly down and upregulated, respectively, in the tomato ripening mutants *rin*/*rin, nor*/*nor*, and *Nr*/*Nr* compared to the wild-type. Authors concluded that ethylene, directly or in combination with the transcription factor *SlMADS-RIN*, regulates the *sHSP* transcript expression. Khaksar and Sirikantaramas showed that the auxin response factor (ARF), *DzARF2A*, transactivates ethylene biosynthetic genes. Also, *DzARF2A* expression was higher in fast-ripening durian cultivars and increased in response to auxin treatment. Therefore, DzARF2A was suggested to play an important role in auxin-ethylene crosstalk to regulate the fruit ripening process in durian. Fresno and Munné-Bosch analyzed three hormones (ABA, JA, and SA) that play a role in fruit development and fruit-microbe interactions on sweet cherry exocarp and mesocarp. The fruit's exocarp had significantly higher concentrations of JA and SA than the mesocarp, while ABA content was similar in both tissues. Authors also reported that endophytic microbial colonization was poor but increased with fruit development, while epiphytic fungi, such as *Alternaria* spp., increased in the mesocarp when the exocarp was cracked. Thus, the absence of concentrated levels of JA and SA and high ABA levels could probably stimulate microbial colonization of mesocarp tissues.

Yu et al. investigated the role of nitric oxide (NO)-induced resistance to *Monilinia fructicola* in peach. Exogenous NO enhanced disease resistance via inhibition of S-nitrosoglutathione reductase (GSNOR) expression and enzyme activity. Also, NO and GSNOR inhibitor (N6022) enhanced the expression of systemic-acquired resistance (SAR)-related genes contributing to disease resistance. In tomato, Yao et al. demonstrated that the addition of hydrogen sulfide (H_2_S) to ethylene treatment maintained high chlorophyll, anthocyanin, and starch content during storage, attenuating the gene expression of the *beta*-amylase (*BAM3*) and ethylene-responsive transcription factors. H_2_S affected pigments' metabolism and the transformation of macromolecular to small molecular metabolites. Altogether, H_2_S delayed the ripening and senescence of tomato fruits during storage. García-Pastor et al. found that foliar spray application of salicylates (SA, ASA, or MeSA) produced a higher concentration of phenolics, anthocyanin, and ascorbic acid at harvest and during storage of pomegranate. Remarkably, salicylate treatments increased crop yield, and red color in pomegranate arils.

Cell wall-degrading enzymes play a key role in fruit ripening (Forlani et al., 2019). Wen et al. performed a genome-wide analysis of pectin methylesterase (PME) in tomato and identified 57 non-redundant PME genes. By analyzing gene expression, three new PME genes were suggested to play a role in fruit ripening, where PE1 and PE2 isoforms could be related to pectin structure at cell junctions and fruit softening. Nakano et al. studied post-harvest characteristics of two ultra-late maturing peach cultivars and found that the cultivar “Daijumitsuto” (DJ) did not soften at all during 3 weeks of storage in response to endogenous and exogenous ethylene, compared to the normal melting flesh (MF) peach cultivar “Tobihaku” (TH). DNA-seq analysis demonstrated that the tandem endo-polygalacturonase (*endoPG*) genes *Pp-endoPGM* (*PGM*) and *Pp-endoPGF* (*PGF*) were deleted in the DJ cultivar, confirming that the *endoPG* genes at *melting flesh* (*M*) locus are responsible for controlling flesh texture in the ultra-late maturing cultivars. Furthermore, genomic analysis of the TH cultivar revealed that an unidentified *M* haplotype (*M*^0^) is the common haplotype in MF peach accessions.

Song et al. analyzed the changes in the redox and antioxidant system in white, pink, and red stages of strawberry fruit development through proteomics analyses using LC-MS and multiple reaction monitoring (MRM) systems. Authors reported novel significant quantitative proteomic changes in antioxidant enzymes (46 proteins and isoforms) during ripening, suggesting that strawberry fruit ripening activates the antioxidant enzymes of a superoxide dismutase (SOD)/glutathione metabolism system.

Tomato ripening mutants SPL-CNR, NAC-NOR, and MADS-RIN were comprehensively characterized at physiological, molecular, and genetical levels during fruit development and ripening by Adaskaveg et al. Through gene expression analysis and direct measurement of hormones, authors found that *Cnr, nor*, and *rin* have alterations in the metabolism and signaling of plant hormones. Remarkably, *Cnr* mutants produce more than basal levels of ethylene, while *nor* and *rin* accumulate high concentrations of ABA. The homozygous *Cnr/nor* double mutant has a *Cnr* phenotype but displayed inhibition of ripening-related gene expression just like *nor* fruit. The fruit trait data generated in this study could be applied to improve the quality and inhibit ripening of tomato hybrids or at least identify tradeoffs between fruit traits.

Finally, two review articles are also included in this Research Topic. Hewitt and Dhingra reviewed the role of the alternative oxidase (AOX) respiratory pathway in mediating cross-talk between ethylene response, carbon metabolism, ATP production, and ROS signaling during climacteric ripening and provided perspectives in post-harvest ripening regulation by AOX. Durán-Soria et al. addressed the role of sugar and its associated molecular network with hormones in the regulation of the accumulation of health-promoting pigments such as anthocyanins and carotenoids both in climacteric and non-climacteric fruit.

Altogether, this Research Topic gathered new information and reviewed the scientific literature on the regulation of fruit ripening at the genetic, transcriptional, proteomic, hormonal, and metabolic levels and their impact on fruit quality. These data provided new insights that could be converted to future applications to improve fruit quality and reduce post-harvest fruit loss (Shipman et al., 2021).

## Author Contributions

CF wrote the first draft of the manuscript and performed the visualization. All authors contributed to conception of the Research Topic, manuscript revision, editing, and approved the submitted version.

## Conflict of Interest

The authors declare that the research was conducted in the absence of any commercial or financial relationships that could be construed as a potential conflict of interest.

## Publisher's Note

All claims expressed in this article are solely those of the authors and do not necessarily represent those of their affiliated organizations, or those of the publisher, the editors and the reviewers. Any product that may be evaluated in this article, or claim that may be made by its manufacturer, is not guaranteed or endorsed by the publisher.
